# Sequential Neural Processes in Abacus Mental Addition: An EEG and fMRI Case Study

**DOI:** 10.1371/journal.pone.0036410

**Published:** 2012-05-04

**Authors:** Yixuan Ku, Bo Hong, Wenjing Zhou, Mark Bodner, Yong-Di Zhou

**Affiliations:** 1 The Key Lab of Brain Functional Genomics (MOE), Institute of Cognitive Neuroscience, School of Psychology and Cognitive Science, East China Normal University, Shanghai, China; 2 Department of Biomedical Engineering, School of Medicine, Tsinghua University, Beijing, China; 3 MIND Research Institute, Santa Ana, California, United States of America; 4 Department of Neurosurgery, Johns Hopkins University, Baltimore, Maryland, United States of America; Hangzhou Normal University, China

## Abstract

Abacus experts are able to mentally calculate multi-digit numbers rapidly. Some behavioral and neuroimaging studies have suggested a visuospatial and visuomotor strategy during abacus mental calculation. However, no study up to now has attempted to dissociate temporally the visuospatial neural process from the visuomotor neural process during abacus mental calculation. In the present study, an abacus expert performed the mental addition tasks (8-digit and 4-digit addends presented in visual or auditory modes) swiftly and accurately. The 100% correct rates in this expert’s task performance were significantly higher than those of ordinary subjects performing 1-digit and 2-digit addition tasks. ERPs, EEG source localizations, and fMRI results taken together suggested visuospatial and visuomotor processes were sequentially arranged during the abacus mental addition with visual addends and could be dissociated from each other temporally. The visuospatial transformation of the numbers, in which the superior parietal lobule was most likely involved, might occur first (around 380 ms) after the onset of the stimuli. The visuomotor processing, in which the superior/middle frontal gyri were most likely involved, might occur later (around 440 ms). Meanwhile, fMRI results suggested that neural networks involved in the abacus mental addition with auditory stimuli were similar to those in the visual abacus mental addition. The most prominently activated brain areas in both conditions included the bilateral superior parietal lobules (BA 7) and bilateral middle frontal gyri (BA 6). These results suggest a supra-modal brain network in abacus mental addition, which may develop from normal mental calculation networks.

## Introduction

The abacus has been used in Asia since 1200 A.D. for rapid precise calculation [1]. The designation of “Abacus experts” refers to those who have an unusual capability of operating a physical abacus for calculation, as well as calculating mentally with an abacus in mind through long-time training [2]. They can swiftly and accurately perform mental addition with large numbers.

There are substantial studies on the neural mechanisms of mental calculation for average people [3], [4], [5], [6], [7], [8]. Some of the studies have supported a cognitive model in which the numbers in mental arithmetic have two representational systems: a language-based system to store tables of exact arithmetic knowledge, and a language-independent system for quantity manipulation and approximation, which relies on visuospatial networks [5]. Others have however suggested that the basic numerical calculation strategy is finger-counting based representation [8]. In addition, a number of neuroimaging studies on neural networks have shown that parietal and frontal areas are the main cortical areas involved in mental calculation for average people [5], [8], [9], [10], [11], [12]. Nevertheless, none of those studies is able to explain the extraordinary capability of mental calculation for abacus experts.

Early behavioral studies suggested that a “mental abacus” was used by abacus experts [13]. During mental calculation, trained-abacus users (but not abacus experts) moved their fingers as if they had been manipulating a real abacus, and prohibition of this movement or interfering with finger-tapping impaired their performance [2]. It seemed that there was also a “mental abacus” in those trained-abacus users’ minds. In addition, Hatano and Osawa demonstrated that in performance of a delayed match-to-sample task, abacus experts could remember a sequence of 16-digits forward or 14 digits backward, which were used as stimuli in the task [14]. The digit memory of the experts was more interfered with concurrent visual-spatial tasks than with aural-verbal tasks [14]. Performance in mental arithmetic tasks of the experts was also more affected by presentation of abacus images than by human faces or digit numbers [15]. All those behavioral studies, together with several neuroimaging studies [16], [17], [18], suggested a visuospatial representation of numbers, which had been developed through abacus practice that involved visuomotor processing under certain rules. It might be more efficient to mentally manipulate multi-digit numbers using a visuospatial representation than a sequentially organized phonological representation [18].

**Figure 1:**
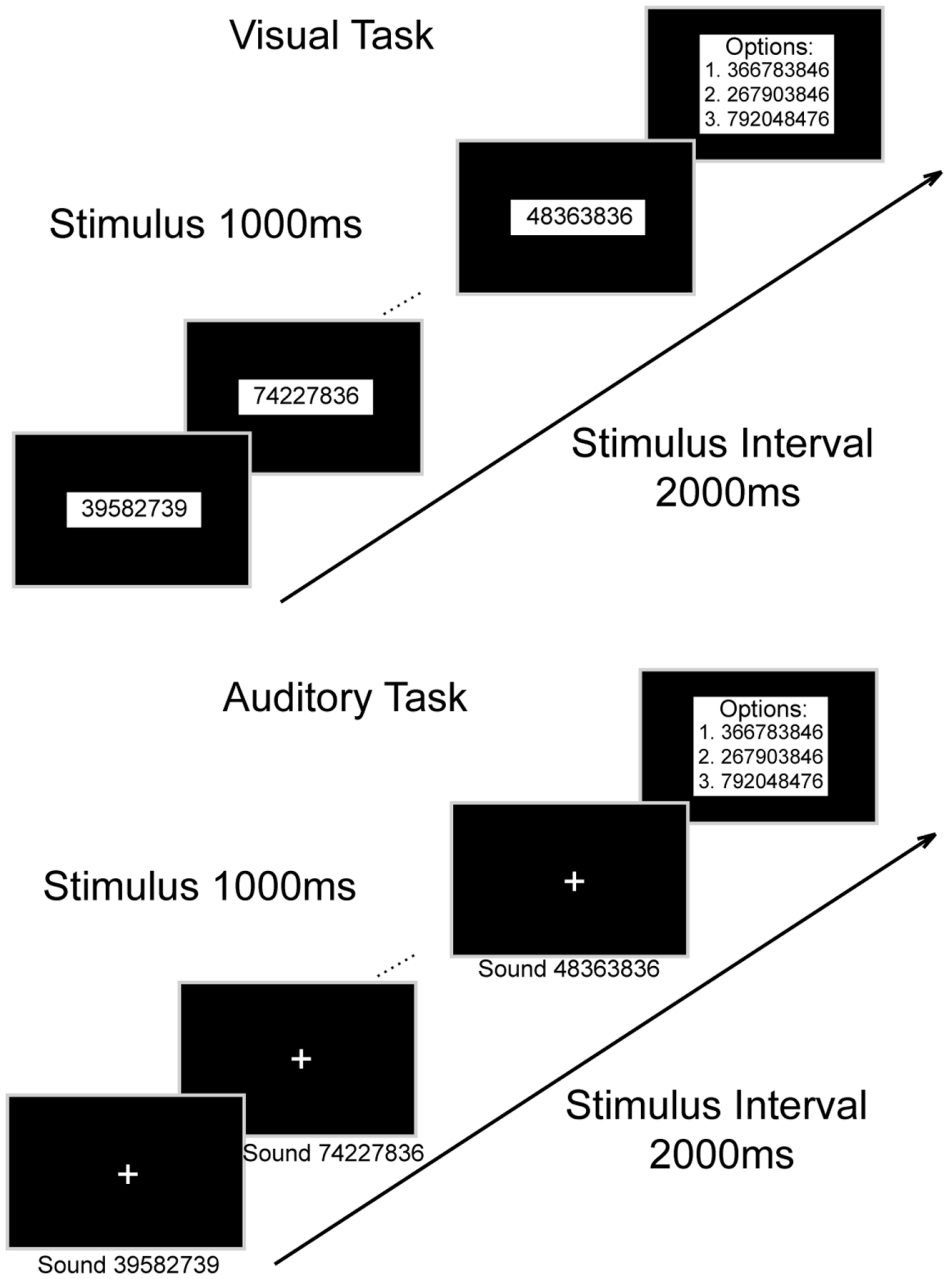
Demonstration of experimental runs in visual and auditory tasks. In visual tasks, the visual stimuli could be 8-digit numbers, 4-digit numbers or 4-letter pseudo-words. Likewise, in auditory tasks, the auditory stimuli could also be 8-digit numbers, 4-digit numbers or 4-letter pseudo-words.

However, none of the above studies had attempted to dissociate the sequential neural processes, one from another, during abacus mental calculation. We thus designed mental addition experiments (see figure 1A and 1B), in which we recorded electroencephalographic (EEG) signals from an abacus expert performing mental addition tasks, in an attempt to examine the temporally sequential processes for mental addition. Furthermore, we measured the subject’s brain activities with functional magnetic resonance imaging (fMRI) when the subject was performing the same tasks, in order to explore the neural network underlying those processes. We hypothesized that there would be sequential visuospatial and visuomotor processes during abacus mental addition, in which both parietal and frontal cortical areas would be involved.

**Figure 2:**
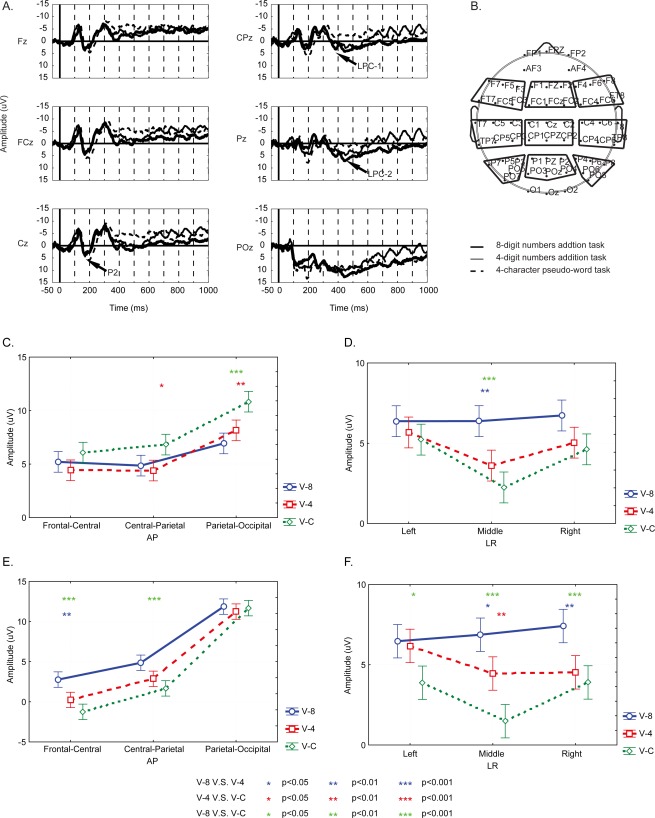
ERP results of the EEG experiments. (A) Grand average of ERPs in visual tasks. The most prominent peaks are named. (B) EEG electrode arrangements. Nine regions of interest (ROI) are displayed with the nine frames. The results of post-hoc Tukey HSD test on the amplitudes of P2 (C), LPC-1 (D, E), and LPC-2 (F).

**Table 1:** Behavioral results from the abacus expert performing the mental addition tasks (8-digit and 4-digit addition), as well as the average subjects performing the mental addition tasks (2-digit and 1-digit addition).

Average subjects	Task	V-2	V-1	A-2	A-1
	AR (%)	77.0±2.5	98.0±0.6	66.3±3.8	96.3±0.9
	RT (s)	1.95±0.14	1.12±0.07	2.30±0.18	1.32±0.14
Abacus expert	Task	V-8	V-4	A-8	A-4
	AR (%)	100	100	100	100
	RT (s)	1.86±0.69	1.65±0.81	2.41±0.91	1.76±0.73

**Table 2:** Statistical results of the three ERP components (* p<0.05; ** p<0.01; *** p<0.001).

	P2 (170∼210 ms)	LPC1 (360∼400 ms)	LPC2 (420∼460 ms)
Amplitude			
TASK	F(2,513) = 20.97***	F(2,513) = 20.03***	F(2,513) = 39.19***
AP	F(2,513) = 46.45***	F(2,513) = 416.06***	F(2,513) = 510.03***
LR	F(2,513) = 9.20***	F(2,513) = 10.08***	F(2,513) = 4.64*
TASK*AP	F(4,513) = 2.64*	F(4,513) = 4.24**	NS
TASK*LR	NS	F(4,513) = 2.56*	F(4,513) = 3.20*
AP*LR	NS	F(4,513) = 13.61***	F(4,513) = 8.75***
TASK*AP*LR	NS	NS	NS
Latency			
TASK	F(2,513) = 5.80**	F(2,513) = 3.90*	NS
AP	F(2,513) = 27.00***	NS	NS
LR	F(2,513) = 23.60***	NS	F(2,513) = 4.20*
TASK*AP	NS	NS	NS
TASK*LR	NS	F(4,513) = 3.80**	NS
AP*LR	F(4,513) = 8.00***	NS	NS
TASK*AP*LR	NS	NS	NS

**Figure 3:**
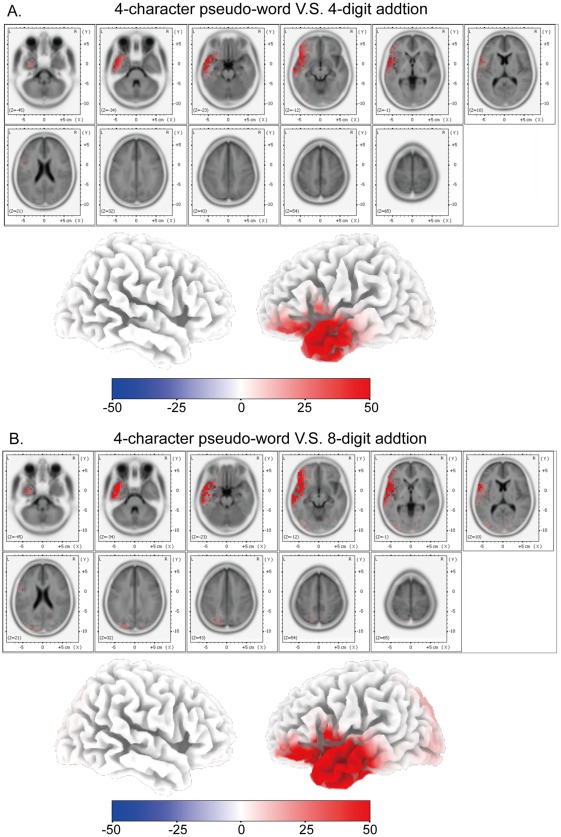
Source localization of P2 component. (A) sLORETA results of the subtraction of P2 traces between V-C and V-4. (B) sLORETA results of the subtraction of P2 traces between V-C and V-8.

**Figure 4:**
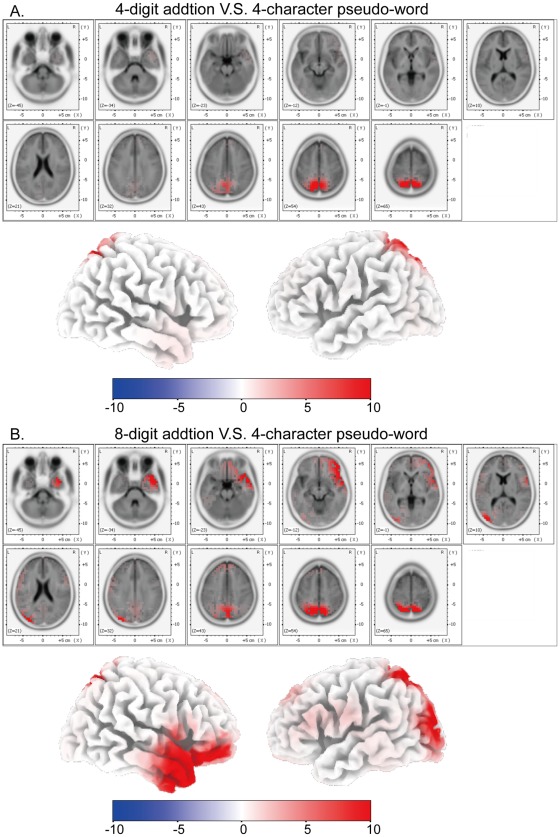
Source localization of LPC-1 component. (A) sLORETA results of the subtraction of LPC-1 traces between V-4 and V-C. (B) sLORETA results of the subtraction of LPC-1 traces between V-8 and V-C.

**Figure 5:**
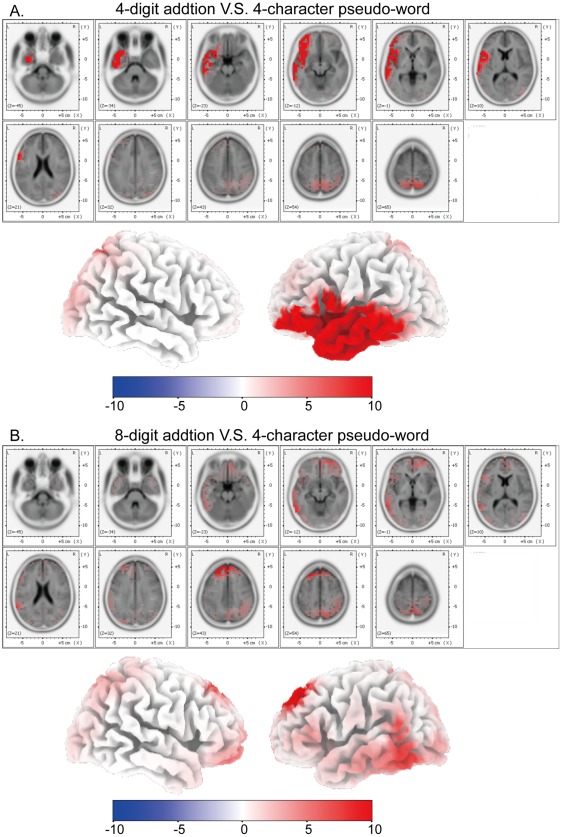
Source localization of LPC-2 component. (A) sLORETA results of the subtraction of LPC-2 traces between V-4 and V-C. (B) sLORETA results of the subtraction of LPC-2 traces between V-8 and V-C.

**Table 3:** The brain areas activated in 4-digit visual mental addition, in contrast to the visual control task (family-wise-error corrected p<0.001).

Anatomical Localization		Coordinates			Cluster (Voxels)	Z-Value
		X (mm)	Y (mm)	Z (mm)		
Superior Parietal Lobule	BA 7	−22	−70	60	577	11.34
	BA 7	−32	−52	56		11.17
Superior Parietal Lobule	BA 7	18	−64	52	676	9.46
	BA 7	34	−46	48		8.89
	BA 7	36	−54	52		7.98
Middle Frontal Gyrus	BA 6	26	−4	50	135	8.45
Middle Frontal Gyrus	BA 6	−22	−14	54	147	8.11
Inferior Parietal Lobule	BA 40	42	−28	40	86	7.87
Inferior Parietal Lobule	BA 40	−50	−40	50	60	6.98
Inferior Frontal Gyrus	BA 9	−58	14	28	54	6.79
Precentral Gyrus	BA 4	−54	−14	26	47	6.67
Inferior Frontal Gyrus	BA 45	54	14	20	38	5.82

**Figure 6:**
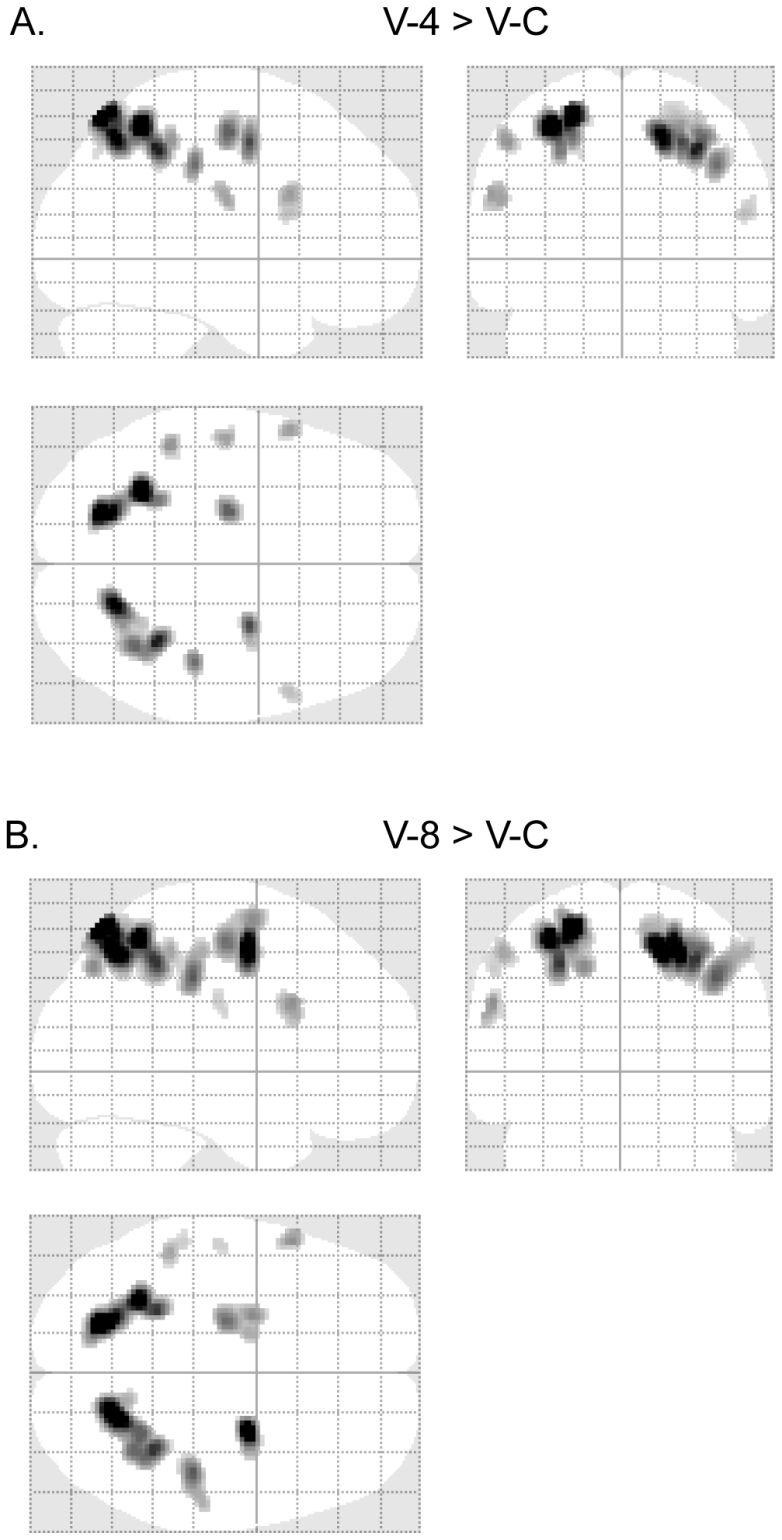
fMRI results in visual mental addition. (A) Glass-brain views of fMRI activated areas of V-4 vs. V-C (p<0.05, family-wise-error corrected). Talairach coordinates of the activated points are listed in table 3. (B) Glass-brain views of fMRI activated areas of V-8 vs. V-C (p<0.05, family-wise-error corrected). Talairach coordinates of the activated points are listed in table 4.

**Table 4:** The brain areas activated in 8-digit visual mental addition, in contrast to the visual control task (family-wise-error corrected p<0.001).

Anatomical Localization		Coordinates			Cluster (Voxels)	Z-Value
		X (mm)	Y (mm)	Z (mm)		
Superior Parietal Lobule	BA 7	−22	−70	60	835	13.95
	BA 7	−30	−52	56		12.38
	BA 7	−24	−62	58		11.1
Superior Parietal Lobule	BA 7	16	−64	54	916	13.1
	BA 7	32	−46	48		10.15
	BA 7	26	−52	52		9.5
Sub-Gyral	BA 6	26	−4	52	278	12.34
Inferior Parietal Lobule	BA 40	42	−28	40	253	9.57
	BA 2	52	−26	50		7.02
Middle Frontal Gyrus	BA 6	−22	−14	56	362	9.19
	BA 6	−24	−2	64		8.16
	BA 6	−16	−2	56		6.93
Middle Frontal Gyrus	BA 9	−56	16	30	74	7.93
Inferior Parietal Lobule	BA 40	−52	−38	50	81	7.22
	BA 40	−56	−32	44		5.74
Postcentral Gyrus	BA 2	−54	−18	30	21	5.65

**Table 5:** The brain areas activated in 4-digit auditory mental addition, in contrast to the auditory control task (family-wise-error corrected p<0.001).

Anatomical Localization		Coordinates			Cluster (Voxels)	Z-Value
		X (mm)	Y (mm)	Z (mm)		
Superior Parietal Lobule	BA 7	−22	−70	60	398	10.17
	BA 7	−30	−52	56		8.32
Superior Parietal Lobule	BA 7	18	−62	50	421	9.48
	BA 7	34	−46	48		6.94
	BA 7	34	−54	54		6.43
Middle Frontal Gyrus	BA 6	26	−4	50	77	7.09
Precentral Gyrus	BA 6	−24	−14	54	100	6.98
Inferior Frontal Gyrus	BA 9	−58	14	28	25	6.3
Inferior Parietal Lobule	BA 40	40	−28	40	15	5.75
Inferior Parietal Lobule	BA 40	−50	−40	50	12	5.75

**Figure 7:**
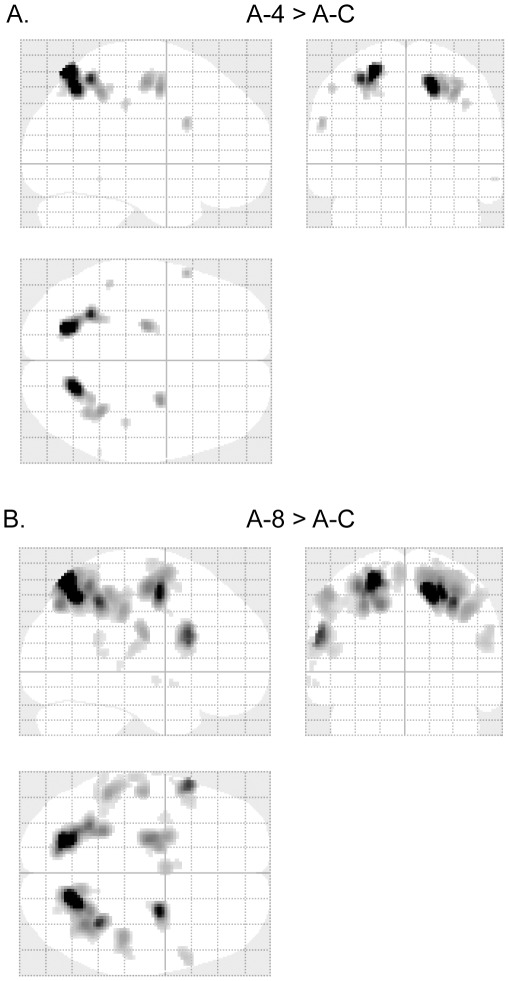
fMRI results in auditory mental addition. (A) Glass-brain views of fMRI activated areas of A-4 vs. A–C (p<0.05, family-wise-error corrected). Talairach coordinates of the activated points are listed in table 5. (B) Glass-brain views of fMRI activated areas of A-8 vs. A–C (p<0.05, family-wise-error corrected). Talairach coordinates of the activated points are listed in table 6.

**Table 6:** The brain areas activated in 8-digit auditory mental addition, in contrast to the auditory control task (family-wise-error corrected p<0.001).

Anatomical Localization		Coordinates			Cluster (Voxels)	Z-Value
		X (mm)	Y (mm)	Z (mm)		
Superior Parietal Lobule	BA 7	−22	−70	60	1308	17.09
	BA 7	−28	−60	52		11.4
	BA 7	−32	−52	56		11.36
Superior Parietal Lobule	BA 7	16	−64	54	1763	15.87
Inferior Parietal Lobule	BA 40	34	−46	46		12.46
	BA 7	34	−54	54		10.79
Sub-Gyral	BA 6	24	−4	54	422	13.45
		22	−8	74		5.98
Inferior Frontal Gyrus	BA 9	−58	16	24	328	12.29
	BA 44	−50	16	14		6.58
Middle Frontal Gyrus	BA 6	−22	−10	56	513	10.93
Superior Frontal Gyrus	BA 6	−24	0	64		9.44
Inferior Parietal Lobule	BA 40	−52	−38	50	348	9.37
	BA 40	−42	−42	46		5.71
Inferior Parietal Lobule	BA 40	44	−28	42	237	9.37
Precentral Gyrus	BA 4	−54	−14	24	174	8.96
Postcentral Gyrus	BA 40	−64	−20	18		6.5
Superior Frontal Gyrus	BA 6	−4	0	66	141	7.81
	BA 6	−6	8	56		5.73
Inferior Frontal Gyrus	BA 45	56	14	20	124	7.12
Supramarginal Gyrus	BA 40	−52	−46	22	71	5.87
Superior Temporal Gyrus	BA 22	−62	−42	12		5.68
Middle Frontal Gyrus	BA 6	−36	−2	46	35	5.4

**Table 7:** The brain areas activated in 8-digit auditory mental addition, in contrast to the 4-digit auditory mental addition.

Anatomical Localization		Coordinates			Cluster (Voxels)	Z-Value	p-Value (FWE corrected)
		X (mm)	Y (mm)	Z (mm)			
Inferior Frontal Gyrus	BA 44	−58	18	24	137	6.00	p<0.001
		−50	16	14		5.70	p<0.001
Inferior Parietal Lobule	BA 40	−52	−34	38	24	5.73	p<0.001
Superior Parietal Lobule	BA 7	−20	−72	58	19	5.65	p<0.01
Superior Temporal Gyrus	BA 22	−60	−44	12	14	5.61	p<0.01
Superior Frontal Gyrus	BA 6	−4	0	66	20	5.58	p<0.01
Superior Frontal Gyrus	BA 6	−24	2	64	18	5.43	p<0.01
Supramarginal Gyrus	BA 40	−52	−46	22	33	5.41	p<0.01

**Figure 8:**
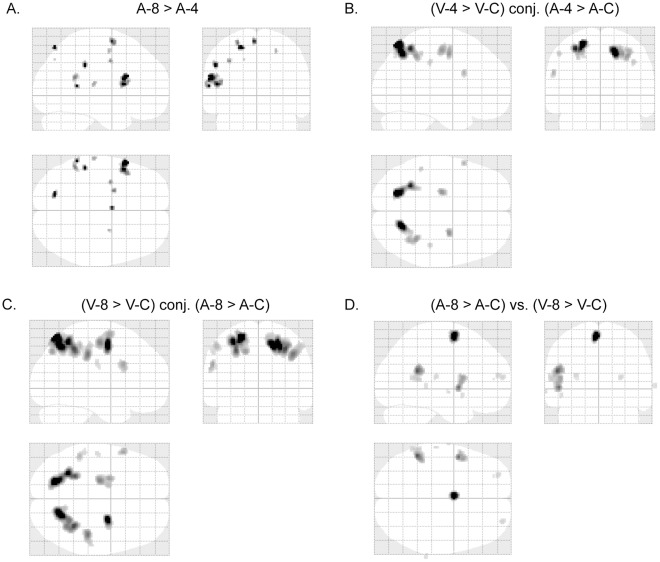
fMRI results revealing difficulty and modality effects. (A) Glass-brain views of fMRI activated areas of A-8 vs. A-4 (p<0.05, family-wise-error corrected). Talairach coordinates of the activated points are listed in table 7. (B) Glass-brain views of fMRI activation conjunction between V4/VC and A4/AC (p<0.05, family-wise-error corrected). (C) Glass-brain views of fMRI activation conjunction between V8/VC and A8/AC (p<0.05, family-wise-error corrected). (D) Glass-brain views of fMRI activated areas of A8/AC vs. V8/VC (p<0.05, family-wise-error corrected). Talairach coordinates of the activated points are listed in table 8.

**Table 8:** The brain areas activated in A8/AC (A-8 in contrast to A-C), in contrast to V8/VC (V-8 in contrast to V-C).

Anatomical Localization		Coordinates			Cluster (Voxels)	Z-Value	p-Value (FWE corrected)
		X (mm)	Y (mm)	Z (mm)			
Superior Frontal Gyrus	BA 6	−2	0	64	151	8.46	p<0.001
Supramarginal Gyrus	BA 40	−52	−46	22	221	6.81	p<0.001
		−60	−46	12		5.46	p<0.01
Inferior Frontal Gyrus	BA 44	−50	6	2	187	6.63	p<0.001
		−48	10	14		6.09	p<0.001
		−56	8	16		5.68	p<0.001
Superior Frontal Gyrus	BA 10	28	62	14	19	5.24	p<0.01
Superior Frontal Gyrus	BA 10	−28	56	16	20	5.20	p<0.05
Middle Temporal Gyrus	BA 22	72	−36	4	11	5.05	p<0.05

## Methods

The protocol of the experiment was approved by the institutional ethics committee in the School of Medicine, Tsinghua University. One abacus expert (female, age 16), who was one of the top performers in the World Mental Calculation Competitions, participated in the experiment as the subject. The subject and her parents gave written informed consents before the experiment.

### Behavioral Tasks for EEG Recording

The EEG experiment was conducted in a dark, sound-proof room, free of ambient electromagnetic interference. The subject was placed in a comfortable chair facing an LED screen at eye level with two speakers, one on each side of the screen, while performing the tasks. Six different tasks (labeled V-8, V-4, V-C, A-8, A-4, and A-C, see descriptions later) were used in this study. Three of them (V-8, V-4, and V-C) were visual tasks, while the other three (A-8, A-4, and A-C) were auditory tasks. The experiment is illustrated in figure 1A and 1B. In the visual tasks, 10 numbers (for mental addition) or 10 pseudo-words (for control) were displayed one by one sequentially in the center of the screen in each experimental run. In the auditory tasks, 10 numbers (or pseudo-words) were pronounced also one by one sequentially through speakers. The duration of each stimulus was one second, and the interval between stimulus onsets was two seconds. In visual tasks, 8-digit numbers (V-8), 4-digit numbers (V-4) and 4-letter pseudo-words (V-C) were presented. In the A-8 task, the 1-s duration of the stimulus was equally divided into eight segments (125 ms for each). Each segment started with an oral presentation of a digit (in Chinese). This digit was randomly selected from nine digits (1 to 9) recorded by a male and its duration was artificially compressed into 100 ms. In A-4 and A-C tasks, the 1-s duration of the stimulus was equally divided into four segments (250 ms for each). Each segment started with an orally presented digit lasting for 230 ms in A-4, and a letter lasting for 230 ms in A-C. In both tasks, the digits and letters were also recorded by a male and artificially compressed into 230 ms. The subject was instructed to maintain focus on the center of the screen in the visual tasks. In the auditory tasks, a cross was presented in the center of the screen during the entire period of a run. The subject was required to fix her sights on the cross. In the mental addition tasks, the subject was required to add the ten numbers presented in succession and then select the correct answer (sum) from three options presented on the screen one second after the off-set of the last of those ten numbers, by pressing one of the corresponding buttons on a keyboard. In the control tasks, the subject was instructed to randomly press one of the three buttons one second after the off-set of the last pseudo-word. Twenty runs were carried out for each task and were grouped into two blocks. The blocks of different tasks were presented randomly. The interval between runs was 3∼5 seconds, and between blocks, 5∼10 minutes. The subject was instructed to avoid unnecessary eye movements during the whole task.

### EEG Recording and Processing

EEG signals were recorded by an EEG recording system (SynAmps2, Compumedics, Ltd Corp, US). Sixty Ag-AgCl scalp electrodes (Quick-Caps, Neuroscan) were arranged in a standard 10–20 system (figure 2B). EEG signals from each electrode were referenced to linked earlobes. Two linked electrodes were placed on the earlobes (one on each earlobe). The signal from the linked earlobe electrodes served as the reference. The impedance of each electrode was kept below 5 kΩ during the experiment. Additionally, the electro-oculogram (EOG) signal was recorded to detect horizontal and vertical eye movements. EEG (60 electrodes) and EOG (2 bipolar electrodes) signals were filtered (0.1–100 Hz band-pass), amplified, digitized (1000 Hz sample rate), and stored for off-line analysis.

EOG contamination in EEG was excluded by means of linear regression implemented in Neuroscan EDIT software (Compumedics, US). Afterwards, the 60 channel EEG data were analyzed using Matlab 7.0 (Math Works, US) and EEGLAB 6.0 toolbox (Swartz Center for Computational Neurosciences, US) [19]. Event-related potentials (ERPs) were calculated by averaging the trials. The baseline was established by averaging voltages from the 50 ms preceding the onset of the stimulus and was subtracted from the ERPs. ERP components were defined as peaks and troughs, and named as routines (P2, LPC-1, and LPC-2 as in figure 2A). The amplitude of an ERP component was measured from its peak to the baseline (peak-value minus the baseline-value). The latency was measured from the stimulus onset to the peak.

### Statistical Analyses

Three-way analysis of variance (ANOVA) was used to compare ERP components (amplitude and latency). Since the offset of an auditory stimulus (an aurally-presented number, or word) could not be defined as accurately as the visual one, only visual ERP components were included in statistical analysis. The electrodes were grouped into 9 regions of interest (ROI) as indicated in figure 2B, and ERPs from electrodes of each region were averaged across electrodes to improve signal-to-noise-ratio. The ANOVA factors were TASK (V-8, V-4 and V-C), AP (frontal-central, central-parietal and parietal-occipital), and LR (left, medial and right). All ERP statistical analyses were performed with Statistica (StatSoft, US).

### sLORETA Analyses

Brain generators associated with modulation of ERPs were estimated by standardized low resolution brain electromagnetic tomographic analysis (sLORETA). The current density was represented in 3-D Tailarach space of the scalp-recorded electrical activity using the sLORETA software package [20]. sLORETA was used to provide an approximate three-dimensional solution of the EEG inverse problem, aiming to determine the most active brain regions in a given instant of time. This analysis was based on the measurements of a dense grid of electrodes, which were placed on the entire scalp surface covering the brain [20].

sLORETA was used to estimate the current source density distribution for epochs of brain electrical activity on a dense grid of 6239 voxels at 5 mm spatial resolution. Three periods were taken for analysis according to the three prominent ERP components: P2, 170–210 ms; LPC-1, 360–400 ms; LPC-2, 420–460 ms. The difference in ERP waveform between the addition task and the control task were submitted for sLORETA [21].

### fMRI Experiments and Data Analyses

The fMRI experiment was carried out inside a three-Tesla MR scanner (Siemens Trio) in the Institute of Biophysics, Chinese Academy of Science. A high resolution T1-weighted multi-sliced anatomical image was acquired in the sagital orientation, using a 3D gradient echo sequence (pulse repetition = 2530 ms; echo time = 3.37 ms; inversion time = 1100; number of signals averaged = 1; matrix size = 256×256×190), for the reference of functional images. Multi-sliced T2*-weighted fMRI images were obtained with a gradient echo-planar sequence using axial slice orientation (25 slices; voxel size = 3.44 mm×3.44 mm×5 mm; matrix size = 64×64×25; TR = 2000 ms; TE = 30 ms; flip angle = 90°). The subject was scanned while performing the task, lying in the scanner and faced with an LED screen where task images (numbers, pseudo-words, and instructions) were projected with an optical fiber. The visual angle was 0.6 degree for each digit or letter in the stimulus. Auditory stimuli were presented through an air-driven earphone system. The subject selected the correct answer by pressing one of the three buttons in a button case. The tasks used in the fMRI experiment were identical to those in the EEG experiment. The fMRI experiment for the subject included four sessions, each of them containing six task blocks (corresponding to six tasks respectively: V-8, V-4, V-C, A-8, A-4, and A-C) that were arranged in a random sequence. Each of those blocks included two experimental runs, and each run consisted of 10 stimuli, corresponding to 10 MRI scans. Therefore, each of the six tasks contained 80 scans in total. The subject took a rest of 10 seconds between blocks and a rest of 3–5 minutes between sessions.

Functional volumes were analyzed by using Statistical Parametric Mapping 5 (SPM5; www.fil.ion.ucl.ac.uk/spm/software/spm5) implemented in MATLAB. Motion correction to the first functional scan was performed using a six-parameter rigid-body transformation [22]. The motion corrected images were normalized to the Montreal Neurological Institute (MNI) coordinate system, and spatially smoothed with a 6-mm full-width-at-half-maximum-isotropic Gaussian kernel. Statistical fMRI analysis was performed using the general linear model (GLM), as implemented in SPM5. Next, we created contrast images for each stimulus between the mental addition task and the control task. Family-wise-error (FWE) was corrected for multiple spatial comparisons. Clusters with more than 10 contiguous supra-threshold (FWE p<0.05) voxels were shown in activation tables.

## Results

### Behavioral Results

Behavioral results of the abacus expert was recorded and listed (table 1) together with the results of our previous study [23] from average subjects who performed addition tasks with fewer digits (1-digit and 2-digit). In all addition tasks, the expert made 100 percent correct choices. The 100% accuracy of the expert was over three standard deviations away from the mean correct rate of the average subjects in each task as given in table 1.

### ERP Results

Averaged event related potentials (ERPs) along the midline electrodes were shown in figure 2A. The three separated ERP components were P2 (peaked around 190 ms), LPC1 (peaked around 380 ms), and LPC2 (peaked around 440 ms). ANOVA results of these ERPs are shown in table 2. Task conditions (TASK: V-8, V-4, V-C) exerted significant effects on amplitudes of all the three ERP components, as well as AP (frontal-central, central-parietal and parietal-occipital) and LR (left, middle, and right) factors. The TASK*AP interaction was significant for the amplitudes of P2 and LPC-1. The TASK*LR interaction was significant for the amplitudes of LPC-1 and LPC-2. Results of the post-hoc Tukey HSD test on the amplitudes of ERP components (P2, LPC-1, LPC-2) are shown in figures 2C–2F. The P2 amplitude was significantly larger in V-C than that in V-8 and V-4 at parietal-occipital recording sites (figure 2C). The LPC-1 amplitude in V-8 was significantly larger than those in V-4 and V-C at middle frontal-central and central-parietal recording sites (figures 2D and 2E). The difference between V-8 and V-C in LPC-1 in the right recording sites was near significance (p = 0.058). The LPC-2 amplitude in V-8 and V-4 was significantly larger than that in V-C at middle recording sites. Meanwhile, the LPC-2 amplitude in V-8 was larger than that in V-4 at middle and right recording sites (figure 2F).

### sLORETA Results

sLORETA was applied to subtractions of ERP traces [21] between the addition task and the control task. Time windows for the analysis were the periods of the three ERP components (P2, LPC-1 and LPC-2). The sLORETA results of these components were shown in figures 3, 4, and 5 respectively. Cortical areas showing maximal activity were observed as follows: around 190 ms (P2), the most active area was located in the left middle temporal gyrus (BA 21) for subtractions between V-C and V-8/V-4 (figure 3); around 380 ms (LPC-1), the most active areas were located in bilateral superior parietal lobules (BA 7) and precuneus (BA 7) for subtractions between V-8/V-4 and V-C (figure 4); around 440 ms (LPC-2), for a subtraction between V-4 and V-C, the most active area was located in the left middle temporal gyrus (BA 21), and for a subtraction between V-8 and V-C, the most active areas were located in bilateral superior frontal gyri (BA 8) and bilateral middle frontal gyri (BA 6) (figure 5).

### fMRI Results

Brain activation of V-4 in contrast to V-C (abbreviation, V4/VC) is shown in table 3 and figure 6A. The most active areas include bilateral superior parietal lobules (BA 7) (cluster size: *left* 577 volumes, *right* 676 volumes; FWE p<0.001) and bilateral middle frontal gyri (BA 6) (cluster size: *left* 147 volumes, *right* 135 volumes; FWE p<0.001). Brain activation of V-8 in contrast to V-C (abbreviation, V8/VC) is shown in table 4 and figure 6B. The most active areas also include bilateral superior parietal lobules (BA 7) (cluster size: *left* 835 volumes, *right* 916 volumes; FWE p<0.001) and bilateral middle frontal gyri (BA 6) (cluster size: *left* 362 volumes, *right* 278 volumes; FWE p<0.001), and the corresponding active cluster sizes are larger than those in table 3 and figure 6A.

Brain activation of A-4 in contrast to A-C (abbreviation, A4/AC) is shown in table 5 and figure 7A. The most active areas include bilateral superior parietal lobules (BA 7) (cluster size: *left* 398 volumes, *right* 421 volumes; FWE p<0.001) and bilateral middle frontal gyri (BA 6) (cluster size: *left* 100 volumes, *right* 77 volumes; FWE p<0.001). Brain activation of A-8 in contrast to A-C (abbreviation, A8/AC) is shown in table 6 and figure 7B. The most active areas also include bilateral superior parietal lobules (BA 7) (cluster size: *left* 1308 volumes, *right* 1763 volumes; FWE p<0.001) and bilateral middle frontal gyri (BA 6) (cluster size: *left* 513 volumes, *right* 422 volumes; FWE p<0.001), and the corresponding active cluster sizes are larger than those in table 5 and figure 7A.

Brain activation of V-8 in contrast to V-4 yields no significant (FWE p<0.05) results. Brain activation of A-8 in contrast to A-4 is shown in table 7 and figure 8A. The most prominent activation is observed in left inferior frontal gyrus (BA 44) (cluster size = 137 volumes, FWE p<0.001).

The conjunction of brain activations between V4/VC and A4/AC is shown in figure 8B, which is a subset of the conjunction of brain activations between V8/VC and A8/AC (figure 8C). The most prominent conjunction activations between V4/VC and A4/AC are observed in bilateral superior parietal lobules (BA 7) (cluster size: *left* 388 volumes, *right* 403 volumes; FWE p<0.001) and bilateral middle frontal gyri (BA 6) (cluster size: *left* 90 volumes, *right* 76 volumes; FWE p<0.001).

Brain activation of V4/VC in contrast to A4/AC yields no significant (FWE p<0.05) results. Brain activation of A8/AC in contrast to V8/VC is shown in table 8 and figure 8D. The most prominent activations are observed in left superior frontal gyrus (BA 6) (cluster size = 151 volumes, FWE p<0.001), left supramarginal gyrus (BA40) (cluster size = 221 volumes, FWE p<0.001), and left inferior frontal gyrus (BA44) (cluster size = 187 volumes, FWE p<0.001).

## Discussion

### Behavioral Results

In the current study, the abacus expert performed the addition tasks with multi-digit (8-digit and 4-digit) numbers swiftly and accurately. Her accuracy rates (100%) were significantly higher than those of the average subjects performing analogous 1-digit and 2-digit addition tasks. The accuracy of the average people performing addition tasks dropped as the addends got larger (more digits in the tasks), which dropped to almost zero as the addends reached 4-digits [24]. Apparently, it is because of years of training in abacus calculation that the expert can perform those tasks quickly and accurately, which are very difficult for average people.

### Sequential Processes During Abacus Mental Addition

The most significant difference observed between the mental addition task and the control task was in the ERP components (P2, LPC-1 and LPC-2). P2 has been considered to be an ERP index of task relevance evaluation of visual stimuli [25]. From this point of view, the P2 in the addition task should be different from that in the control task. Indeed, the P2 amplitude in the addition condition was significantly lower than that in the control condition. The source of the difference was located most prominently in the left middle temporal gyrus as shown in the sLORETA results. The middle temporal gyrus is considered to play an important role in visual perception [26], [27], which further explained why there was a difference in the P2 component between the addition condition and the control condition. Meanwhile, the left middle temporal gyrus has been shown to be activated during associative semantics [28]. We therefore assume that the semantic factor also plays a role in perception of the stimuli.

LPC-1 and LPC-2 were two late components. Previous studies revealed that LPC-1, peaked around 380 ms, and was involved in mental calculation for ordinary subjects [7], [23], [29]. The LPC-1 for the normal subjects showed a calculation-dependent difference between the mental calculation task [V-2 (visual 2-digit mental addition) or V-1 (visual 1-digit mental addition)] and the control task [7], [23], [29]. However, this component did not show any difficulty effect between V-2 and V-1 [23]. If neural processes for abacus mental addition are similar to those for mental addition of normal subjects, the LPC-1 amplitude in both situations may show similar effects. That is, the LPC-1 amplitude in V-8 and V-4 would be larger than that in V-C, and the amplitude in V-8 would be similar to that in V-4. However, in this study, the LPC-1 amplitude in V-8 is significantly higher than those in the other two tasks (V-4 & V-C as shown in figure 2E and 2D), and no significant difference in LPC-1 has been observed between V-4 and V-C. These results indicate that neural processes for mental calculation of the expert are not as same as those of ordinary subjects. As suggested by many behavioral and neuroimaging studies on abacus mental calculation, a visuospatial imagery strategy might be taken by experts [2], [14], [15], [16], [17], [18], which was different from a phonological strategy in mental calculation used by ordinary people [5], [23]. The sLORETA results revealed that the source of the difference in LPC-1 between the mental addition task and the control task was maximally located bilaterally in the superior parietal lobule (figure 4). Superior parietal lobule is important in processing visuospatial information [30], [31], [32], [33]. Taken together, the LPC-1 component might therefore represent transformation of the visually presented digits into an abacus image, since for trained-abacus users, the interruption of an abacus image could disturb their mental calculation [15]. We also observed activation of the right middle temporal gyrus (figure 4B) in the sLORETA results, which may contribute to the right-lateralized ERP difference in frontal-central areas (figures 2E and 2D).

The LPC-2 amplitude in the addition task was significantly larger than that in the control task. Meanwhile, the LPC-2 amplitude in V-8 was significantly larger than that in V-4 (figure 2F). The sLORETA results revealed that the most prominently activated sources, which resulted in the difference in the LPC-2 between V-8 and V-C, were located in bilateral superior frontal gyri (BA 8) and bilateral middle frontal gyri (BA 6), while the difference in the LPC-2 between V-4 and V-C, was located in the left middle temporal gyrus (BA 21) (as shown in figure 5B, BA8 and BA6 were also activated in this analysis as shown in figure 5A, although not the most prominently activated). The superior frontal gyrus and the middle frontal gyrus play an important role in visuomotor process [34], [35], [36]. Thus, the visuospatial information transformed in the previous stage represented by LPC-1 might be further processed, represented by LPC-2, in those frontal areas through mental abacus manipulation that we assumed as a visuomotor process to get the intermediate results.

FMRI results in the visual tasks (as in tables 3 and 4 and figure 6) showed that compared with activated brain areas in the visual control task, the most prominently activated areas in the visual mental addition task included bilateral superior parietal lobules (BA 7) and bilateral middle frontal gyri (BA 6). These fMRI results were consistent with our sLORETA results, and further validate the sLORETA analysis.

### Difficulty Effects and Modality Effects

Difficulty effects on the amplitude of LPC-1 and LPC-2 were observed between V-8 and V-4. sLORETA analysis revealed that sources of the difference were located in bilateral superior parietal lobules and bilateral middle frontal gyri. We found that the cluster size of those cortical areas in V8/VC were larger than V4/VC in fMRI results, although no significant activations were observed at the level FWE p<0.05. However, if the significant level is set to uncorrected p<0.001, the activation of those areas would also be observed. These results indicate that the network involved in abacus mental calculation is more activated with an increase of task difficulty.

FMRI results in the auditory tasks (tables 5 and 6 and figure 7) are similar to those in the visual tasks. In both tasks, the most prominently activated areas are bilateral superior parietal lobules (BA 7) and bilateral middle frontal gyri (BA 6). Further conjunction analysis was performed between V4/VC and A4/AC (figure 8B). Besides BA 7 and BA 6, the activated areas also included bilateral inferior parietal lobules, and left inferior frontal gyrus. All these conjunction areas belonged to a subset of conjunction activations between V8/VC and A8/AC (figure 8C). These results suggested that the abacus mental calculation for both visual and auditory stimuli shared similar brain networks.

The most prominent activated areas observed with A-8 in contrast to A-4 (table 7 and figure 8A) are left superior frontal gyrus, left supramarginal gyrus, and left inferior frontal gyrus. These areas have been suggested to be phonologically and linguistically related areas [37], [38]. The phonological difference between stimuli from A-8 and A-4 respectively may result in activations of these cortical areas. Similar results were observed in contrasting A8/AC to V8/VC (table 8 and figure 8D).

### Working Memory and Mental Calculation

Baddeley proposed a model of working memory with a central executive and two slave systems of the visuospatial sketchpad and the phonological loop [39]. Many studies have investigated the relationship between the sub-components of working memory and mental calculation. Most of those studies examined the role of central executive and the phonological loop in mental calculation [40], [41], [42], [43], [44], [45]. However, very few studies have examined the role of visuospatial sketchpad in mental calculation, although Dehaene et al. suggested that the visuospatial strategy might be important for approximate mental calculation [5].

The results of EEG and fMRI from the abacus expert in the current study suggest the presence of sequential visuospatial and visuomotor processes. Furthermore, behavioral performance of the expert is far better than that of the average people in our study, which suggest a different strategy for task performance has been taken by the expert (likely using a mental abacus). The visuospatial sketchpad might play a critical role in abacus mental calculation.

### From the View of Neuronal Recycling

The “neuronal recycling” hypothesis emphasizes that cultural acquisitions must take place within the limited surface and bounded plasticity of the human cortex [46]. In our study, the abacus expert, through long time training, had developed an extraordinary ability to do addition with multi-digit numbers quickly and accurately. This tremendous capability should also be based on the existing cortical structure. Some brain areas, such as the superior and inferior parietal lobules, and the middle and inferior frontal gyri, have been suggested to be correlated with mental arithmetic [5], [8], [9], [10], [11], [12], [47], [48]. Activation of these areas was also observed in our study (as in tables 3, 4, 5, and 6). One developmental study has revealed a process of increased functional specialization of inferior parietal cortex in mental arithmetic [48]. The involvement of bilateral inferior parietal lobules in our study indicates that the abacus mental arithmetic may have developed from the normal mental arithmetic. Nevertheless, the abacus mental calculation is different from that of the normal mental calculation. Some different strategies may have been taken during abacus mental calculation as indicated by the broader activation of visuospatial and visuomotor brain areas observed in our study. One recent diffusion tensor imaging (DTI) study also suggested this difference between the abacus user group and the control group by showing higher average fractional anisotropy in left occipitotemporal junction and right premotor projection in the abacus group [49].

### Summary

In this study, we used behavioral tests as well as EEG and fMRI techniques to explore the abacus mental calculation process. The abacus expert could quickly and accurately perform mental addition tasks with multi-digit numbers, which was not possible for the average people to complete. The EEG and fMRI results revealed sequential visuospatial and visuomotor processes during abacus mental addition. Superior parietal lobule might take part in the process of visuospatial transformation around 380 ms after the onset of the stimuli. Superior/middle frontal gyrus might participate in the visuomotor process around 440 ms. Abacus mental addition with both visual and auditory stimuli seems to share similar brain networks, mainly involving bilateral superior parietal lobules and bilateral middle frontal gyri.
